# Silence Among First-Line Managers in Eldercare and Their Continuous Improvement Work During Covid-19

**DOI:** 10.1177/00469580221107052

**Published:** 2022-07-26

**Authors:** Lotta Dellve, Mimmi Kheddache Jendeby

**Affiliations:** 1 University of Gothenburg, Göteborg, Sweden

**Keywords:** employee silence, leadership, communication, eldercare, continuous improvement work

## Abstract

Eldercare sector faced severe needs, and unexplained difficulties, to manage daily work and the continuous improvement of routines at operative levels during Covid-19. First-line managers in eldercare have a key role to facilitate learnings but may be hindered in public, hierarchical organizations. This is the first study on the conditions and importance of silence for managerial work in terms of daily operations and continuous improvement work. To identify first-line managers’ silence in eldercare, its contextual and supportive conditions, its reasons and its implications for managerial work with regard to daily operations and continuous improvement work. Mixed-method study based on a questionnaire to first-line managers (n = 189) in Swedish public eldercare in 33 randomly selected municipal organizations and one city. The instruments Communication of Critical Issues at Work, Managers Stress Inventory and Managerial Work and an open question were analyzed using: (1) qualitative coding to explore organizational conditions, (2) descriptive statistics, and (3) stepwise regressions to identify associations. The most common forms of silence were quiescent (based on fear of the consequences of speaking up) and acquiescent (based on resignation and demotivation). Organizational conditions shaping managerial silence were due to strict governance and control in a hierarchical organization, lack of support and participation in decision-making and the experience of not being valued. Managers’ silence had a negative impact on managerial work and especially work on continuous improvements. The pandemic also offered space for values of occupational professionalism and learning at operational levels. Organizational conditions of support through superiors and management teams decreased silence. Manager silence is detrimental for continuous improvement work and may arise in organizations with dominant values of organizational professionalism. Supportive conditions based on trust and space for occupational professionalism may be important and should be improved to decrease managerial silence and better support continuous improvements.

**What do we already know about this topic?**
Employee silence at operational levels is a key obstacle for development work in health care organizations. Studies of managerial silence and, silence in eldercare are rare. The huge needs of development of routines in eldercare was highlighted during pandemic.
**How does your research contribute to the field?**
By identification of first-line managers’ silence and its strong importance for continuous improvement work at operative levels of eldercare. Managerial silence was lower where support through superiors, management teams and employees were stronger. Dominating values of organizational professionalism was indicated key processes behind managerial silence.
**What are your research’s implications toward theory, practice, or policy?**
The results suggests improvements of supportive organizational conditions to operative managers and also to balance organizational versus occupational professional values in organization.

## Introduction

Despite decades of systematic and continuous improvement work in health care,^1^ there are great gaps in such efforts in eldercare. This was highlighted in Sweden and other European countries during the Covid-19 pandemic. News media and assessments by government agencies showed severe deficiencies regarding safety for the old adults and neglected continuous improvements of hygiene routines and other work processes of importance for quality and safety.^2^ Debates were initiated concerning how we value the elderly in Swedish society and other European countries and whether there is a culture of silence preventing employees and managers from communicating about misconduct.

Although a few studies have looked at the relationship between voice, individual factors and employment conditions in eldercare,^3-5^ no study has thus far focused on silence among managers. In public eldercare organizations with distributed management of practice, the lower-level managers have a key role in leading the continuous improvement work.^6^ The present study departs from theories on occupational and organizational professionalism^7^ to form a basis of how organizational conditions may shape first-line managers’ (FLMs) silence and work in eldercare. The aim is to identify first-line managers’ silence in eldercare, its contextual and supportive conditions, its reasons and its implications for managerial work with regard to daily operations and continuous improvement work. The following questions guided the analysis: what organizational contextual conditions are experienced among FLMs, shaping silence? What kind of silence are prevalent among FLMs in eldercare? The 2 hypothesis tested, were: (1) FLMs who experienced support within organization have a lower degree of silence; (2) Lower degree of silence will have a positive effect on FLMs work with daily operations and continuous improvement work.

## Background

### Organizational Conditions for Silence

Silence among employees can be understood from an individual as well as an organizational perspective.^8^ It is thus important to understand both the organizational context affecting the employee as well as individual reasons for remaining silent.

Morrison and Milliken^8^ describe a set of conditions—such as an economic and financial focus, high level of cost control, great power distance and steep hierarchies as well as centralized decision-making—which are hypothesized to create a breeding ground for silence in organizations. These conditions have been described as manifesting a new sort of professionalism having grown stronger in the knowledge-based service sector, namely organizational professionalism.^7^ The ideal type of organizational professionalism represent values such as control, hierarchical structures of responsibility, decision-making, and standardization.^7^ In eldercare, organizational professionalism coexists with occupational professionalism. The ideal type of occupational professionalism relies on values such as knowledge, autonomy, discretion, and assessment.^7,9^ In reality, managers in eldercare are likely to experience manifestations of these forms of professionalism to varying degrees. If the values of organizational professionalism becomes too dominant in organizations, it will risk creating conditions that may undermine voice and lead to silence.^8^ It has moreover been found in practice that when employees experience top managers to act in accordance with values of organizational professionalism this can foster a silence culture.^10^ For example, New Public Management with its focus on efficiency, standardization, and measurement supports organizational professionalism more than occupational professionalism.^7^ This governance principle had a tight grip on the Swedish public sector for 3 decades and is still apparent even when public organizations are moving toward more trust-based governance and management.^11,12^

### Managerial Work

FLMs play a key role in creating conditions for active development work.^6^ In eldercare, their role often encompasses implementing quality management in practice, bridging gaps between strategic and operational functions, cooperating with development managers and creating conditions for employees to engage in development work.^13^ The work of managers is often under scrutiny both internally and externally.^14^ Despite their high demands and responsibilities, FLMs are often alone in their role, and they express a need of support from their superiors and employees to be able to conduct and create conditions for communicating daily operations, errors and development work.^15,16^ The strict control and lack of support in communicating challenges and deficiencies could create fear, resignation, and reduced transparency (silence).^14^ This could potentially result in a paradox where managers have to uphold a flawless facade and at the same time accommodate democratic values and principles of transparency.

The actions of top management may affect the willingness of FLMs to share information^17^ by supporting open communication, building trust, and promoting continuous improvements at lower levels.^18-20^ Studies from healthcare organizations and eldercare also show that supportive relationships at lower levels can have a positive impact on reporting errors.^21-23^

### Silence and Continuous Improvement Work

Silence in organizations could undermine organizational development^8^ as it results in missed opportunities to share important information and learn from mistakes. Continuous improvement work relies on identifying gaps between a desired outcome and the actual outcome in order to trigger a learning process where the root cause of the discrepancy is communicated, analyzed, and managed.^24^

Being able to both manage ongoing work and carry out continuous improvement work became even more important during the corona crises in the eldercare sector. The implications of the new virus created complicated and complex learning situations as these implications included a large number of unknown variables and sometimes implied having to question underlying assumptions and beliefs in the organization. Argyris and Schön^24^ refer to this more difficult and emotionally charged learning situation as double-loop learning. The corona crises probably triggered a large number of double-loop learning situations where employees in eldercare had to question and challenge their ability to bridge different values, such as sharing information in a transparent manner and being perceived as a high-quality institution. The presence of more defensive values, such as wanting to be in unilateral control or avoiding negative feelings, means that double-loop learning becomes difficult, as these values have a negative effect on our ability, willingness, or courage to voice concerns regarding challenging situations.

When reacting to a challenging situation, employees can either exit the situation, voice their concern^25^ or become silent and disengage from work.^26^ Pinder and Harlos^27^ define employee silence as “the withholding of any form of genuine expressions about the individuals’ evaluations of his or her organizational circumstances to persons who are perceived to be capable of effecting change or redress” (p. 334). Employee silence is thus not necessarily the same as remaining silent but rather means that the communication does not reflect a desire to change the circumstances or it is not directed at a person able to change those circumstances.^27^ Knoll and van Dick^28^ identify 4 different forms of silence. *Acquiescent silence* implies that the employee has lost hope in terms of being able to influence the situation. It is an expression of demotivation or resignation, when the employee feels that his or her opinions are not wanted nor valued. *Quiescent silence* is when employees refrain from expressing their views to protect themselves as they fear consequences. *Prosocial silence* concerns protecting and benefiting others, refraining from expressing one’s opinions so that co-workers, managers or the organization will not get hurt or are benefited. It could moreover be an expression of tolerance—not wanting to make a fuss. *Opportunistic silence* implies that the employee wants to gain benefits from not expressing his or her view; for instance, avoiding extra work.^28^ All these forms of silence, albeit stemming from different motivational states, limit the sharing of potentially valid information, thus reducing the ability to manage the working conditions and develop the work and the organization.^29^

## Methodology

### Study Design

To identify first-line managers’ silence in eldercare, a questionnaire was distributed to first-line managers in (a) 33 randomly selected organizations and (b) one large city in Sweden. This selection was made in order to have a sample representing organizational conditions among different types of municipalities in Sweden. To explore contextual conditions shaping managerial silence, responses to an open-ended question were analyzed qualitatively. The quantitative part assesses organizational conditions and identifies their importance for managers’ silence and managerial work with regard to daily operations and continuous improvement work. Ethical approval was obtained from the Ethics Committee (Dnr: 2019-02934).

### Study Setting

The study was conducted in Sweden and is based on questionnaires distributed to FLMs working in eldercare. For the past 2 decades, Swedish municipalities are responsible for organizing eldercare. Legislation and national rights related to social security and employment regulations look the same in all municipalities. However, how municipalities organize the provision of care varies, as do local working conditions. For instance, municipalities differ in terms of both structural, demographic and financial conditions.

In the Swedish public debate, poor quality and a lack of patient safety in eldercare have been in focus since April 2020. This has led to more attention from the government and municipalities, such as through inspections from government agencies pointing to poor safety conditions as well as poor conditions for first-line managers in terms of managing their work.^2^ The work of being a first-line manager comprises responsibility for the service rendered, working conditions and budget. All employees are required to report abuse or risks of abuse (Lex Sarah, 2001:453, chapter 14, 3§) to the Health and Social Care Inspectorate.

### Study Sample and Data Collection

In order to reflect various managerial conditions, a random selection was made of 33 municipalities out of Sweden’s 290 municipalities. The selected municipalities were geographically situated in all parts of the country. They included municipalities from 8 of the 9 classifications of municipalities, which are made on the basis of structural parameters such as population and commuting patterns. The random selection did not include any large cities. Thus, we also selected one of Sweden’s three large cities and, for practical reasons, the one closest to us.

In the selected municipalities, managers of eldercare were identified through websites and direct contact with an administrator. A questionnaire was distributed via personal email from May to June 2021 to 472 first-line managers, 189 of whom responded (40% response rate). About 40% managed eldercare in ordinary living and 60% in home-like residential facilities. Among the responding managers, women make up the largest portion of the sample (88%). Further, the vast majority of respondents had undergone post-secondary education (96%) and/or some sort of management training (95%). The mean number of employees per manager was 35.6 (md = 32), and the average time working as a manager (in total) was approximately 12 years (md = 11).

### Managerial Silence

Items from the instrument *Communication of Critical Issues at Work*^28^ were translated from English into Swedish and back. The instrument starts with the introduction of the concept: “From time to time, employees face problematic situations at work. For example, they think that colleagues or supervisors act in a way that is wrong, inefficient, immoral, or otherwise problematic. People deal differently with such situations; that is, some voice their concerns and try to change the situation, whereas others remain silent. We are interested in whether you noticed such a problematic situation at work and whether you spoke up to someone who can change the situation or tended to remain silent.” Respondents are then asked to rate how often they expressed concerns or opinions to someone capable of changing the situation or whether they remained silent. Individuals who remained silent were asked to consider a range of statements regarding the reasons for their silence: *Acquiescent silence* (Cronbach’s α = .77) was constructed from 2 statements: because (1) nothing would have changed anyway; (2) no one would have listened anyway. *Quiescent silence* (Cronbach’s α = .76) was constructed from 2 statements: because of (1) bad experiences I have had when speaking up on critical issues in the past; (2) fear of negative consequences. *Prosocial silence* was assessed using a single-item statement: because I did not want to embarrass others. *Opportunistic silence* was assessed using a single-item statement: because that would have led to avoidable additional work. Responses were given on seven-point Likert scales ranging from 1 “Never” to 7 “Very frequently.” The face validity of the items was tested among 6 public sector managers.

### Managerial Work

Systematic managerial work at the unit was assessed using the Systematic Occupational Health and Safety Management Questionnaire.^30^ *Managerial work—daily operations* was assessed using 2 items: (1) Are you satisfied with your ability to fulfill your responsibilities regarding daily work in a trustful and safe manner at your unit; and (2) To what extent do you think that the daily work in the unit you are responsible for has improved over the last 6 months? (Cronbach’s α = .51). *Managerial work—continuous improvement work* was assessed using 4 items. Two of the questions began with: Are you satisfied with your ability to fulfill your responsibilities regarding improvement work in a trustful and safe manner at your unit? Two of the questions began: To what extent do you think that the improvement work at the unit you are responsible for has improved over the last 6 months? In both items, responses were given both regarding continuous daily improvement work and the long-term developments (Cronbach’s α = .85).

### Organizational Conditions

*Support through superiors* (3 items, Cronbach’s α = .85) through *Management team* (2 items, Cronbach’s α = .60) and through *Employees* (4 items, Cronbach’s α = .89) as well as *Group dynamic challenges* (4 items, Cronbach’s α = .85) and *Strict governance and control* (3 items, Cronbach’s α = .76) belong to validated indices retrieved from the Gothenburg Manager Stress Inventory (GMSI).^31^ In addition, *information* (top-down) regarding ongoing changes was assessed using a single item. For these items, respondents are asked to assess aspects of their own work situation during the past 6 months on a scale from 1 “Not at all true” to 5 “Completely true.” *Dialog with employee at unit* (3 items, Cronbach’s α = .75) was assessed using 3 items pertaining to transparent and open communication between managers and subordinates regarding their active work with improvements in terms of user safety and quality of care.^32^ On a Likert scale ranging from 1 “Not at all true” to 5 “Completely true,” respondents were asked to evaluate the extent to which a range of statements were true at the unit/organization for which they are responsible.

*Open question*: Managers could freely present their point of view immediately following the items concerning silence: *Finally, you have the opportunity to, in your own words, describe what you have experienced and view as important regarding organization, the work of leaders and organizational conditions during the pandemic.* Answers from 80 managers consisting of a total of about 4000 words were analyzed.

### Analysis

First, a descriptive analysis of managerial silence (mean, SD, and proportion) was performed. Second, bivariate regressions were analyzed between managerial silence and organizational conditions. Third, a multivariate analysis was conducted, stepwise and forward, to test the hypothesis. Model 1 stepwise included the kinds of silence exhibiting statistical significance. Model 2 kept the kinds of silence and stepwise included the organizational conditions exhibiting statistical significance. The data from the open question was analyzed using content analysis.^33^ First, an initial, line-by-line coding was conducted, based on theories on occupational and organizational professionalism. Focus coding was then applied and reoccurring patterns were thematized with the aim of capturing contextual and supportive conditions. Examples of themes include “lack of support,” “experience of pressure,” and “what a manager needs to have and be.” The themes were then analyzed through the concept of silence. Last, we investigated it there were major differences between the city and the other municipality regarding main findings (eg, qualitative themes, mean values).

## Qualitative Findings Regarding Conditions Shaping Silence

### Harboring Values of Organizational Professionalism

Demands originating from values of organizational professionalism in a hierarchical organization were experienced by managers, analyzed as creating conditions for silence. Managers described a lack of support from superior managers, lack of participation in decision-making, not being valued and experiences of within-organization distance that undermined trust and psychological safety. Factors that could all create breeding ground for silence. Managers expressed feelings of being left alone and not being considered and cared for.

*Decisions that are made but not established together with managers or employees. Budget work that is more important than anything.*

*Why aren’t people, managers, co-workers and users important in our organization?*

*First-line managers have once more become quite bad and expensive garbagemen/administrators and are not fully seen as a resource to achieve results, to develop or succeed in the management by objectives process.*

*One tends to forget that managers too have a work environment; above all, first-line managers working in two directions.*

Different sorts of pressures in terms of strict control and a focus on costs were experienced; in particular, economic pressures related to budgetary control also when factors were not under their control and administrative pressure due to delegated responsibilities for budget, control of services and measuring results. These pressures, originating from demands of control related to values of organizational professionalism, can undermine voice and create conditions for silence as they signal a lack of trust.

*I have participated in countless meetings about finances where I am supposed to deliver action plans to improve the negative result due to the virus and cohort care and high sick leave rates. Events that I cannot affect.*

*We were on our knees when it came to getting access to fill-ins, and here, I didn’t experience that we received more money, instead we were supposed to stick to the budget.*

*Eldercare has become a disaster when it comes to administration. Too much time is spent on documenting one thing after another.*

The managers experienced organizational conditions based on demands to harbor the values of organizational professionalism that could foster managerial silence. They experienced a clash between the values of organizational professionalism and the values of occupational professionalism. Such a clash was also related to feeling strained and exhausted, which got worse due to the pandemic.

*Very difficult with recovery the last year. I am worn out as a manager.*

### Increased Space for Values of an Occupational Professionalism and Trust

The significance of relationships for preserving the managers’ occupational professionalism in a large and highly structured hierarchical organization was clearly emphasized during the pandemic. The increased closeness with the employees and the focus on the mission implied shared learning processes and a broadened acceptance of changes. Managers also experienced positive emotions regarding what they accomplished and gratitude toward their employees. These factors play a key role with regard to occupational professionalism, which the pandemic also gave more space for due to canceled meetings.

*The most important thing in my leadership is to be close to the operations, to be together with the staff during my workday.*

*I still think that the co-workers have done an extremely good job as we had three weeks in May last year with pandemic. After that, we managed to keep it away with strict routines and hygiene controls as well as information to users and their relatives.*

*Covid-19 has taught me a lot about my leadership and enabled me to be there at work instead of traveling around the city for different meetings that don’t concern the core mission – I have had time to focus on the right things.*

*Despite a lack of other forms of competence improvement, we have never ever learned so much at such a fast rate as now. This has implied a quick change in the operations with the biggest acceptance of change that I have ever experienced.*

The managers shared a desire to move toward more trust-based conditions. They expressed occupational values such as focusing on the old adults and improved communication to better take learning and experiences into account. The qualitative data points to that there are organizational conditions in eldercare that could create a breeding ground for silence and that values have partly been at odds during the pandemic.

*Communication the whole way up to the management team and the politicians and the whole way down to the co-workers needs to be better.*

*In order to find the right way, administrations need to work toward trust-based organizations and stop shaping managers into old-fashioned “unit supervisors” and instead recruit transformative leaders.*

*Trust is the alpha and the omega of a good and safe work environment.*

*I therefore consider it to be of utmost importance that experience from practical work should be taken into account when making decisions.*

## Quantitative Results

### Silence Among First-Line Managers

The results from the quantitative analysis identified the prevalence and variation in silence among operational managers in municipal organizations (Table 1). Many (41%) were often silent. The main forms of silence were due to being afraid (quiescent silence, 37% of whom experienced this more often than occasionally) and being resigned (acquiescent silence, 32%). Opportunistic silence and prosocial silence were rated to a higher degree among 28% and 10% of the FLMs, respectively. Managers responsible for more employees (>30) rated a higher level of all types of silence. Managers with longer experience rated a higher level of acquiescent and quiescent silence. Managers in homecare rated a higher level of opportunistic silence and a lower level of prosocial silence.

**Table 1.** Descriptive Data of Kinds of Silence and Managers’ Work and Organizational Conditions.

	Silence			
	Acquiescent mean (SD)	Quiescent mean (SD)	Prosocial mean (SD)	Opportunistic mean (SD)
All	3.89 (1.52)	4.08 (1.86)	3.24 (1.51)	3.22 (1.99)
Homecare	4.0 (1.69)	4.15 (1.96)	2.5 (1.26)	3.92 (1.97)
Number of employees <30	3.60 (1.39)	3.81 (1.90)	2.96 (1.29)	2.75 (1.94)
>30	4.29 (1.64)	4.41 (1.83)	3.67 (1.74)	3.81 (1.94)
Years of experience as manager <10	3.90 (1.50)	3.84 (2.02)	3.20 (1.58)	3.26 (1.88)
>10	3.88 (1.58)	4.32 (1.72)	3.27 (1.45)	3.17 (2.16)

The different reasons for silence showed various correlations with organizational conditions of support and communication in collaboration with superiors or subordinates (Table 2). The strongest associations were typically found for acquiescent and quiescent silence. Further, the positive associations with regard to supportive superiors and management teams—and negative with regard to strict governance and control—were stronger than support through employees and group dynamic challenges.

**Table 2.** Bivariate Correlations Between Kinds of Silence and Organizational Conditions of Support and Communication.

	Silence			
	Acquiescent	Quiescent	Prosocial	Opportunistic
Kind of silence				
Acquiescent	1			
Quiescent	0.49	1		
Prosocial	0.32	0.24	1	
Opportunistic	0.49	0.21	0.02	1
Organizational conditions				
Support through: Superiors	–0.36	–0.32	–0.17	–0.29
Management team	–0.36	–0.32	–0.07	–0.21
Employees	–0.08	–0.06	–0.01	0.04
Group dynamic challenges	0.27	0.15	0.30	–0.06
Strict governance and control	0.40	0.45	0.27	0.31
Information (top–down)	–0.31	–0.26	–0.31	–0.36
Dialog with employees at unit	–0.03	–0.21	0.15	–0.30

### Importance of Silence for Continuous Improvement Work

Almost one-third (28%) were satisfied with their work and improvements of the daily work, while 24% were not satisfied at all, and their managerial work had deteriorated in this respect. Regarding continuous improvement work, one-third (34%) were satisfied and experienced improvements, while 13% rated their managerial work as having deteriorated. The multivariate analysis of associations between silence and managerial work was conducted stepwise (Table 3). Model 1 includes kinds of silence. Model 2 includes statistically significant organizational conditions. The analysis showed the importance of silence for managerial work with regard to daily operations and improvements, also when supportive organizational conditions were included.

**Table 3.** Stepwise Regression Models Including (1) Kinds of Silence and (2) Kinds of Silence and Organizational Conditions of Support and Communication.

	Managerial work			
	Daily operations		Improvement work	
	Model 1 beta (*P*-value)	Model 2 beta (*P*-value)	Model 1 beta (*P*-value)	Model 2 beta (*P*-value)
*Model 1 Managerial silence*				
Quiescent	–0.19 (.02)	–0.18 (.02)		
Opportunistic	–0.23 (.00)	–0.23 (.01)	–0.31 (.00)	–0.21 (.00)
*Model 2 + Org. conditions*				
Support from superior				0.28 (.01)
Support through employees		0.41 (.05)		
Dialog at unit				0.42 (.00)
*R* ^2^	.39	.46	.39	.70
*R* ^2^ *adjusted*	.34	.41	.37	.67

For managerial work regarding daily operations, quiescent and opportunistic silence exhibited a negative association also when the positively associated supportive collaboration with employees was included. Opportunistic silence exhibited a negative association for managerial work regarding continuous improvement work, also when the positively associated support from superiors and dialogs with employees were included.

The first models where only silence was included explained 39% of the variation in managerial work (*r*^2^). When organizational conditions were included, the second models explained 46% of the variation in managerial work related to daily operations and 70% of continuous improvement work.

## Discussion

The qualitative findings describe organizational demands originating from the values of organizational professionalism as key processes behind managerial silence. Due to a dominance of values of organizational professionalism, their personal and occupational role and voice were perceived as not being valued, and the lower-level managers described feelings of resignation and being left without useful organizational support for their important operational work. This was confirmed in the quantitative analysis by identifying (a) silence among almost half the FLMs with quiescent and acquiescent silence being the most common reasons, (b) a strong association between managerial silence and improvement work, (c) strict governance and control that increased silence and, (d) that a perception of support through superiors and management teams decreased silence. The present study indicates a link between a focus on values of organizational professionalism and a managerial silence perspective at lower operational levels. Thus, the findings relate to theories on employee silence and values of organizational and occupational professionalism. Morrison and Milliken^8^ hypothesize that when top management focuses on control and cost management, the response of silence is more likely. Further, the experience of hierarchical structures focusing on the budget and a large administrative workload represent manifestations of what Evetts refers to as organizational professionalism. Moreover, the values of organizational professionalism, where structures are vital, coexists with values of occupational professionalism, which to a large extent is based on relationships.^7,9^

The results suggest the importance of considering the conditions of lower managers and to balance values. When values of organizational professionalism dominate at the expense of values of trust and occupational professionalism, this may create a basis for FLMs being silent. Silence due to being afraid to give voice or previous negative experiences of giving voice (*quiescent silence*) were more common and also associated with a lower degree of systematic managerial work. The fact that opportunistic silence had a negative effect on daily operations and improvement work even when support from superiors were experienced could potentially have to do with a heavy workload during the corona crisis. That first line managers refrained from voicing concerns due to an already heavy workload would most likely undermine their ability to fulfill responsibilities regarding both daily work and improvement work. Managers facing challenging conditions (ie, in homecare and managers with a large number of employees) indicated a higher level of quiescent silence. This silence was also rated among managers with longer managerial experience and was associated with stricter governance and control framing the managerial duties. These qualities and system structures are known to be more common in organizations in which managers exhibit fears and unproductive beliefs. Morrison and Milliken^8^ suggest that organizations may be characterized by silence as a result of managers’ fears and beliefs concerning other actors in the organization. In their system model, such “silence organizations” view lower-level managers and employees as less competent and willing to do their job. They will thus perceive the act of speaking up as futile or even dangerous. This power distance between higher and lower management may increase the risk of negative consequences for people wishing to speak up to address concerns.^34^ Moreover, when decisions taken at the top of the organizations are very difficult or even impossible to implement further down, there is a significant risk that people at lower levels become less motivated as a result.^35^ Then, acquiescent silence may be an alternative, which was also moderately correlated with quiescent silence. This kind of silence was also rated higher among managers with a large number of employees and among managers in homecare. Additionally, when responsibility is unclear or when FLMs are forced to comply with rigid rules and controls, it is very difficult to further spark a willingness to take responsibility and address challenging situations.^35^ When a superior manager, on the other hand, shows trust by sharing information, opening up for dialog and supporting ownership among the employees, they may trigger reciprocal behavior and a cycle of building trust can be set in motion.^36^ Holland et al found that voice builds trust in senior management and that trust is a mediating factor between a supervisor’s support and the attitudes of employees, especially engagement. Being able to voice concerns sends a signal of being valued, which builds trust.^19^

The pandemic put an end to meetings unnecessary for handling everyday work. This increased the space for operational continuous improvement work through values of occupational professionalism (ie, working closely with employees regarding core issues and continuously learning and improving through their experiences). An organizational climate that allows employees to speak up can have a positive effect on the implementation of new practices^17^ through its important role in creating conditions for trust and engagement.^37^ When managers share information and when feedback is allowed, information will flow more freely, and double-loop learning can take place. Creating a climate in which lower-level managers and employees experience support from their managers is thus very important when creating conditions for voice in an organization as well as when reducing the levels of quiescent and acquiescent silence. Such a climate can help handle ongoing work as well as restore existing practices during a crisis.

### Methodological Concerns

This was the first time that the instrument of employee silence was used in Sweden and among managers. The translation was carried out by a professional language expert and critically examined regarding face validity among 6 managers in the public sector. A strength of the method is the random selection of municipal organizations. The limitations concern that (a) only parts of the instrument could be included, (b) the study is cross-sectional, and (c) a relatively small number of participants due to the low response rate.

## Conclusion

Dominant values of organizational professionalism may form the basis for managerial silence. The silence of FLMs is detrimental for continuous improvement work. The pandemic also offered space for values of occupational professionalism and learning. Supportive conditions based on trust may be important and should be improved to decrease managerial silence.
